# Deviations in gait metrics in patients with chronic ankle instability: a case control study

**DOI:** 10.1186/s13047-014-0058-1

**Published:** 2015-01-21

**Authors:** Roy Gigi, Amir Haim, Elchanan Luger, Ganit Segal, Eyal Melamed, Yiftah Beer, Matityahu Nof, Meir Nyska, Avi Elbaz

**Affiliations:** Department of Orthopedic Surgery, Tel Aviv Sourasky Medical Center, Sackler Faculty of Medicine, Tel Aviv University, 6 Weizmann Street, Tel Aviv, 64239 Israel; AposTherapy Research Group, Herzliya, Israel; Department of Orthopedic Surgery, Rambam Medical Center, Haifa, Israel; Department of Orthopedic Surgery, Assaf Harofeh Medical Center, Zerifin, Israel; Department of Orthopedic Surgery, Meir Medical Center, Kfar-Saba, Israel

**Keywords:** Chronic ankle instability, Gait, Quality of life

## Abstract

**Background:**

Gait metric alterations have been previously reported in patients suffering from chronic ankle instability (CAI). Previous studies of gait in this population have been comprised of relatively small cohorts, and the findings of these studies are not uniform. The objective of the present study was to examine spatiotemporal gait metrics in patients with CAI and examine the relationship between self-reported disease severity and the magnitude of gait abnormalities.

**Methods:**

Forty-four patients with CAI were identified and compared to 53 healthy controls. Patients were evaluated with spatiotemporal gait analysis via a computerized mat and with the Short Form (SF) - 36 health survey.

**Results:**

Patients with CAI were found to walk with approximately 16% slower walking velocity, 9% lower cadence and approximately 7% lower step length. Furthermore, the base of support, during walking, in the CAI group was approximately 43% wider, and the single limb support phase was 3.5% shorter compared to the control group.

All of the SF-36 8-subscales, as well as the SF-36 physical component summary and SF-36 mental component summary, were significantly lower in patients with CAI compared to the control group. Finally, significant correlations were found between most of the objective gait measures and the SF-36 mental component summary and SF-36 physical component summary.

**Conclusions:**

The results outline a gait profile for patients suffering from CAI. Significant differences were found in most spatiotemporal gait metrics. An important finding was a significantly wider base of support. It may be speculated that these gait alterations may reflect a strategy to deal with imbalance and pain. These findings suggest the usefulness of gait metrics, alongside with the use of self-evaluation questionnaires, in assessing disease severity of patients with CAI.

## Background

The definition and classification of chronic ankle instability (CAI) are problematic. Clinically, it is defined as recurrent subjective complaint of the ankle joint “giving way” [1,2] and as “repetitive bouts of lateral ankle instability resulting in numerous ankle sprains” [3]. CAI is usually the sequelae of acute ankle sprain [4], and up to 34% of the patients suffer from a residual problem within the 3 years following their first incident [4]. Some individuals with CAI are limited in participating in sports and even in activities of daily living for years after the initial injury [5,6]. The first insult usually involves hyper-supination (a combination of inversion, plantar flexion, and internal rotation) of the hind foot in relation to the tibia, resulting in injury to the lateral ankle ligaments. The most commonly injured ligaments are the anterior talofibular and the calcaneofibular [7].

The term “mechanical instability” (MI) has been used to describe patients with objective physical examination and radiologic findings (e.g., stress radiographs) suggestive of ligamentous incompetence about the ankle-joint complex. However, there is a low correlation between these findings and long-term prognosis. Freeman et al. [1] coined the term “functional instability” (FI) to describe participants who experience multiple ankle joint inversion injuries with slight or no external provocation yet have no objective evidence of insufficiency of the static ankle stabilizers. Those authors proposed that these injuries are the result of functional instability in ankle strength [8] and proprioception [9-11]. In 2002, Hertel proposed a model involving MI and FI that has become widely accepted [3]. In that model, MI and FI are not considered as being mutually exclusive but rather as part of a continuum, and that recurrent sprain occur when both conditions are present.

Gait deviations have been previously described in CAI patients. They were found to display an inappropriate positioning of the ankle joint before heel strike [12], hesitation towards the end of the stance phase and tendency to put greater load on the lateral forefoot, causing a lateral shift of the foot’s center of pressure [13]. However, most reports on gait metrics comprised relatively small cohorts of CAI patients and the results were inconsistent [12,13]. Furthermore, in a previous study we showed that a simple gait assessment of spatiotemporal parameters may objectively quantify the functional condition of patients with knee osteoarthritis and correlates with the patient’s pain, functional limitation and quality of life perception [14]. To the best of our knowledge, there is missing data regarding the assessment and characterization of spatiotemporal gait of patients with CAI. Therefore, this study had two aims. First, to examine alterations in spatiotemporal gait metrics in patients with CAI compared to healthy participants. We hypothesized that patients with CAI will display deteriorated gait patterns compared to healthy participants. Secondly, to examine the relationship between self-reported disease severity (subjective assessment of the patient) and the magnitude of gait abnormalities (objective assessment). We hypothesized that a correlation will be found between objective gait metrics and subjective assessment of pain and function.

## Methods

Approval from the Institutional Review Board of our Medical Center was obtained before initiating this study. The study was registered in the NIH clinical trial registration system (No. NCT00767780). This study is part of a series of research works that aim to characterize patients with different musculoskeletal disorders compared to healthy controls. The research methodology has been presented previously by Assa et al. [15]. This was a retrospective study of individuals with CAI and healthy individuals who served as control group. Patients with CAI were recruited from AposTherapy center, after seeking treatment for their ankle instability problem. Patients were assessed before treatment and results were used to characterize their gait patterns and symptoms compared to healthy controls. All patients were instructed to refrain from taking pain medications, including paracetamol and non-steroidal anti-inflammatory drugs, for a period of 3 days prior to the clinical and gait evaluation. The control group was comprised of asymptomatic healthy volunteers, mostly employees or caregivers that underwent the same measurements at AposTherapy center. Inclusion criteria for patients with CAI were based on Caulfield and Garrett’s criteria [16,17]: reported recurrent ankle sprains, instability, and a tendency of the ankle to “give way” during sports activities for at least the past 6 months. The inclusion criterion for the control group was the absence of any history of ankle sprains. Exclusion criterion for patients with CAI was a recent injury to the ankle joint (less than 6 months). Exclusion criteria for both patients with CAI and the control group were a history of fracture to the lower extremity, severe systemic diseases, lower extremity orthopedic surgery, infection, concomitant injury from the hip knee or lumbar spine, or history of neurological or vestibular impairments.

The study database was searched for patients diagnosed as having CAI. Between May 2009 and February 2012, 95 patients with CAI were referred to the center, of whom 44 fulfilled the study criteria for CAI. They included 16 women and 28 men with a mean ± SD age of 36.7 ± 15.0 years and a mean ± SD body mass index (BMI) of 25.7 ± 4.6 kg/m^2^. Thirty-five patients had unilateral CAI and 9 patients had bilateral CAI, with one side being more symptomatic. The control group consisted of 53 healthy participants (24 women and 29 men) with a mean ± SD age of (36.6 ± 13.3 years) and a mean ± SD BMI of 23.8 ± 3.3 kg/m^2^ (Table 1). Patients in the control group underwent the same investigative protocol as patients with CAI.

**Table 1:** **Patients’ characteristics**

**Group**	**Males**	**Females**	**Age (y)**	**Height (m)**	**Weight (kg)**	**BMI (kg/m** ^**2**^ **)**
CAI (n = 44) (SD)	64%	36%	36.7 (±15.0)	1.72 (±0.10)	75.7 (±15.2)	25.7 (±4.6)
Control (n = 53) (SD)	54%	46%	36.6 (±13.3)	1.72 (±0.10)	71.4 (±13.6)	23.8 (±3.3)
*P* ^a^	0.374		0.986	0.981	0.095	0.035

^a^ *p*-value was set to *p* < 0.05.

BMI = body mass index; CAI = chronic ankle instability; SD = standard deviation.

### Data acquisition and processing

All data were retrieved from the patients’ medical files and the controls’ records. All patients with CAI underwent a comprehensive assessment during their first visit to the therapy center as well as a physical examination, including manual muscle testing to verify neurological deficits by a certified physical therapist. Anthropometric measurements of height and weight were taken of all participants, and spatiotemporal gait metrics were measured by means of a computerized mat (GaitMat system, E.Q., Inc. Chalfont, PA) [18]. The definition of spatiotemporal gait metrics refers to a group of measures that captures either temporal (for example, velocity) or spatial (for example, step length) gait characteristics. During the gait test patients with CAI and participants in the control group were asked to walk barefoot at a self-selected speed. They walked 3 meters both before and after being tested on the mat to allow sufficient acceleration and deceleration time outside the measurement area. Four trials were conducted, and the acquired data were stored for later analysis. The mean value of the four trials was calculated for each of the following parameters: velocity (cm/s), step length (cm), cadence (steps/min), stride length (cm), base of support (cm), swing (% gait cycle [GC]), stance (%GC), single limb support (%GC) and double limb support (%GC). The latter four measures are representative of the gait cycle phases. Stance phase is when the limb is in contact with the ground whereas the swing phase is when the limb is not in contact with the ground. The stance phase can be divided into two additional phases: single limb support which is the time spent on one limb while the contralateral limb swings forward and, double limb support which is the time when both limbs are in contact with the floor. Left and right double limb support is determined based on the leading limb during heel strike. These measures can be displaced as absolute numbers (i.e. time in seconds) or as a percent of the gait cycle. Furthermore, left and right values are measured for step length, stride length, swing, stance, single limb support and double limb support. The analysis included values of the more symptomatic ankle and less symptomatic ankle. This was determined by the patient’s answer to the questions which ankle is more symptomatic. For the control group, the results of the left limb were defined as the more symptomatic limb and the results of the right limb were defined as the less symptomatic limb included in the analysis.

Differences in perceived pain, function and health-related quality of life (QoL) were evaluated using the SF-36 Health Survey [19]. The SF-36 (version 1) is scored between 0 and 100, with 0 indicating the worst QoL and 100 indicating the best QoL. The physical component summary (PCS) is a summary scale comprised of questions regarding physical functioning, role of limitation due to physical problems, bodily pain and general health (overall 22 questions). The mental component summary (MCS) is a summary scale comprised of questions in the fields of vitality, social functioning, role of limitations due to emotional problems and emotional wellbeing (overall 14 questions).

### Statistical analysis

All statistical analyses were carried out by an independent biostatistician. Mean values and standard deviations were calculated for all continuous measurements. The distributions of gait characteristics and the questionnaire scales were examined using the Kolmogorov–Smirnov non-parametric test, and parametric or non-parametric statistical tests were applied based on the distribution. The main purpose of the study was to compare between the two groups in gait parameters. The sample size for this study had enough power to detect the following differences: velocity (power: 0.99), cadence (power: 0.99), step length (power: 0.85), stride length (power: 0.90), base of support (power: 0.98), swing (power: 0.76), stance (power: 0.76), single limb support (power: 0.92) and double limb support (power: 0.95). The power calculation was done during the statistical analysis of the results phase and not a priori, since we did not have previous data on spatiotemporal measures of patients with CAI. The results of the current study may help in future power calculation analysis of research works on patients with CAI.

A Chi-Square test was used to analyze sex differences between the groups. Non-parametric Mann–Whitney tests were used for comparison of self-reported pain and functional data, comparisons of spatiotemporal parameters between the two groups and for a comparison between patients with unilateral CAI and patients with bilateral CAI. Data for male and female participants in isolation was done in order to examine trends between these subgroups. Statistical significance was accepted for *p* values less than 0.05. The relationships between gait metrics and the SF-36 health survey scores were examined using the Spearman correlation. The suggested cut-off of Dancey and Reidy were used. They suggest that values between 0.1 to 0.3 are considered weak, values between 0.4 and 0.6 are considered moderate, values between 0.7 to 0.9 are considered strong and a value of 1 is considered a perfect correlation [20]. All analyses were performed using SPSS (version 19.0).

## Results

Significant differences in BMI were found between patients with CAI and the control group (*p* = 0.035). However, analysis of the results controlling for BMI as co-variant was performed (using GLM ANCOVA statistic test). Results did not change, i.e. all the significant differences that were found between the groups maintained their trend. Hence, we assumed that although significant, BMI differences between groups did not affect the results and did not explain the differences that were found between groups.

Nine patients (20%) had bilateral CAI and the other 35 patients (80%) had unilateral symptoms. A comparison between the differences in gait patterns of both groups was carried. There were no significant differences in gait patterns between patients with unilateral CAI and patients with bilateral CAI (lowest p-value was *p* = 0.115).

There were significant differences between patients with CAI and the control group in gait velocity, cadence, step length, stride length, single limb support and double limb support (Table 2). These differences maintained when comparison was done for males and females separately. Walking velocity (cm/s) and cadence (steps/min) were significantly lower in patients with CAI relative to the control group (by 19.7 cm/s and 6.9 steps/min, *p* < 0.001, respectively). Step and stride length of the more symptomatic ankle were lower in patients with CAI compared to the control group by (5.0 cm, *p* = 0.003 and 10.8 cm, *p* = 0.002, respectively). A significant reduction in the single limb support of the more symptomatic ankle (38.7% GC versus 40.1% GC, *p* = 0.001) and an increase in the double limb support of the more symptomatic ankle (21.8% GC versus 19.6% GC, *p* < 0.001) were recorded for patients with CAI compared to the control group. There was also a statistically significant increase (2.3 cm) of the width of the base of support in patients with CAI compared to the control group (*p* < 0.001).

**Table 2:** **Gait spatiotemporal metrics differences between patients with CAI and healthy controls**

	**CAI**	**Control**	**Mean difference [CI]**	***p***
Velocity (cm/s)	105.9 (20.0)	125.6 (19.0)	19.7 [11.8 to 27.6]	<0.001
Cadence (steps/min)	69.5 (7.0)	76.4 (5.7)	6.9 [4.3 to 9.4]	<0.001
Step length more symptomatic (cm)	60.8 (8.1)	65.9 (8.1)	5.0 [1.8 to 8.3]	0.003
Step length less symptomatic (cm)	60.3 (9.0)	66.1 (8.1)	5.8 [2.3 to 9.2]	<0.001
Stride length more symptomatic (cm)	121.1 (16.9)	131.9 (16.1)	10.8 [4.1 to 17.4]	0.002
Stride length less symptomatic (cm)	121.3 (16.9)	131.9 (16.1)	10.6 [3.9 to 17.3]	0.002
Base of support (cm)	6.9 (3.5)	4.5 (2.3)	−2.3 [−3.5 to −1.1]	<0.001
Swing more symptomatic (% GC)	39.4 (1.9)	40.3 (1.4)	0.9 [0.2 to 1.6]	0.010
Swing less symptomatic (% GC)	38.7 (2.4)	40.1 (1.7)	1.4 [0.6 to 2.2]	0.001
Stance more symptomatic (% GC)	60.6 (1.9)	59.7 (1.4)	−0.9 [−1.6 to −0.2]	0.010
Stance less symptomatic (% GC)	61.3 (2.4)	59.9 (1.7)	−1.4 [−2.2 to −0.6]	0.001
Single limb support more symptomatic (% GC)	38.7 (2.4)	40.1 (1.6)	1.4 [0.6 to 2.2]	0.001
Single limb support less symptomatic (% GC)	39.4 (1.9)	40.2 (1.5)	0.8 [0.1 to 1.5]	0.022
Double limb support more symptomatic (% GC)	21.8 (3.3)	19.6 (2.6)	−2.3 [−3.5 to −1.1]	<0.001
Double limb support less symptomatic (% GC)	21.9 (3.3)	19.7 (3.0)	−2.2 [−3.5 to −0.9]	0.001

Results are presented as mean (±SD) and 95% CI.

CAI = chronic ankle instability; SD = standard deviation; CI = confidence interval; GC = gait cycle.

The scores on SF-36 bodily pain and SF-36 physical functioning subscales were significantly lower in patients with CAI compared to the control group (*p* < 0.001 for both subscales). Likewise, all other six subscales of the SF-36 health survey, as well as the integrated PCS and MCS, were significantly lower in patients with CAI (*p*-values ranged between *p* = 0.008 to *p* < 0.001) (Table 3). These differences maintained when comparison was done for males and females separately. Spearman’s correlation analysis with gait values as objective measures and subjective pain and functional scores in patients with CAI are presented in Table 4. There was a significant positive correlation between the PCS and gait velocity, step length, stride length, swing, stance and single limb support (*p*-value ranged between 0.003 to 0.048, r-value ranged between 0.300 to 0.442). For example, patients with high PCS scores presented higher walking velocity. There was a significant negative correlation between PCS and double limb support, i.e. patients with high PCS scores had low double limb support scores (r = −0.308, *p* = 0.042 and r = −0.318, *p* = 0.035 for the more symptomatic DLS and less symptomatic DLS, respectively). There was a significant positive correlation between the MCS and gait velocity, step length, swing, stance and single limb support (*p*-value ranged between 0.010 to 0.050, r-value ranged between 0.297 to 0.386). All the correlations that were found significant were within the range of moderate correlation.

**Table 3:** **SF-36 health survey differences between patients with CAI and controls**

	**CAI**	**Control**	**Mean difference [CI]**	***p***
SF-36 (0–100)				
Physical functioning	57.3 (28.4)	95.2 (8.2)	37.9 [29.0 to 46.8]	<0.001
Bodily pain	48.8 (23.4)	90.3 (12.6)	41.5 [33.7 to 49.4]	<0.001
Limitation due to physical problems	34.1 (40.0)	91.5 (21.3)	57.4 [44.0 to 70.8]	<0.001
Limitation due to emotional problems	57.7 (21.0)	70.8 (15.5)	31.7 [16.5 to 46.9]	<0.001
Vitality	67.1 (20.8)	76.9 (12.9)	13.0 [5.4 to 20.6]	0.001
Social functioning	61.4 (46.6)	93.1 (21.0)	23.5 [15.5 to 31.6]	<0.001
Emotional well being	72.4 (26.6)	96.0 (12.0)	9.8 [2.6 to 17.0]	0.008
General health	60.0 (20.6)	80.0 (12.8)	20.0 [12.9 to 27.1]	<0.001
Physical component summary (PCS)	51.6 (20.0)	85.6 (9.7)	34.0 [27.4 to 40.6]	<0.001
Mental component summary (MCS)	63.7 (19.5)	83.4 (11.3)	19.6 [13.0 to 26.3]	<0.001

Results are presented as mean (±SD) and 95% CI.

CAI = chronic ankle instability; CI = confidence interval.

**Table 4:** **Correlation (r) between gait metrics and SF-36 physical component summary and SF-36 mental component summary in patients with CAI**

	**SF-36 Mental component summary**	***P*** ^**a**^	**SF-36 Physical component summary**	***P*** ^**a**^
Velocity (cm/s)	0.349	0.020	0.359	0.017
Cadence (steps/min)	0.297	0.050	0.284	0.062
Step length more symptomatic (cm)	0.241	0.115	0.300	0.048
Step length less symptomatic (cm)	0.308	0.042	0.317	0.036
Stride length more symptomatic (cm)	0.277	0.068	0.314	0.038
Stride length less symptomatic (cm)	0.275	0.071	0.308	0.042
Base of support more symptomatic (cm)	0.025	0.873	−0.038	0.808
Swing more symptomatic (% GC)	−0.103	0.507	0.003	0.986
Swing less symptomatic (% GC)	0.386	0.010	0.442	0.003
Stance more symptomatic (% GC)	0.103	0.507	−0.003	0.986
Stance less symptomatic (% GC)	0.386	0.010	0.442	0.003
Single limb support more symptomatic (% GC)	0.383	0.010	0.423	0.004
Single limb support less symptomatic (% GC)	0.000	0.554	−0.092	0.998
Double limb support more symptomatic (% GC)	−0.218	0.155	−0.308	0.042
Double limb support less symptomatic (% GC)	−0.225	0.142	−0.318	0.035

^a^ *p*-value was set to *p <* 0.05.

## Discussion

The present study evaluated the spatiotemporal gait (objective) parameters and the self-evaluation (subjective) questionnaires in a relatively large cohort of patients with CAI and compared the findings to a healthy control group. The results revealed that gait metrics differ significantly between the two groups.

The diagnosis and evaluation of patients with CAI pose a real clinical challenge. It is difficult to classify a pathology that is primarily subjective in nature. For example, “giving way” of the ankle is often used as an inclusion criterion for studies of CAI [16,17], but “giving way” is subjective and can be either a real occurrence or the patient’s interpretation of something else entirely. Only recently consensus values for selection criteria for patients with CAI were established by the Ankle Consortium Position Statement but there is still a need for more objective measures [21]. The results of the current study present the deviations in objective gait metrics of patients with CAI compared to controls. All patients were diagnosed with CAI and did not have other musculoskeletal problems, therefore it was assumed that the reported differences in gait metrics of patients with CAI compared to healthy controls are indeed due to the patient’s adaptations to ankle instability. Our findings were similar to previous studies that reported the changes in gait patterns of patients with CAI [12,13]. It may be assumed that an objective spatiotemporal gait test may be sensitive enough to detect these changes. However, further studies are needed to determine how spatiotemporal parameters are affected by CAI. Furthermore, future studies should examine how different treatments for CAI affect spatiotemporal parameters by including gait assessment as an outcome measure.

Even though gait alterations are present for days and weeks after acute lateral ankle sprain [22,23], little is known about the long-term alterations in gait of patients with CAI. Moreover, we could not find previous data on the spatiotemporal gait patterns of patients with CAI. Most studies examined kinetic and kinematic changes. For example, Nyska et al. reported a significantly longer ground-contact time of the heel and midfoot areas in patients with CAI compared to a control group [13]. Those authors speculated that this is because patients are hesitant to put weight on the forefoot. Mattson and Brostrom and Ebig et al. suggested that a slowed reflex response time of the peroneal and tibialis anterior muscles to sudden plantar flexion and inversion stress may cause difficulties with ankle joint function for patients with CAI [24,25]. Others noted that local sensory deficits [26] and impaired proprioception [27,28] also contribute to instability, even in the absence of muscle weakness [29,30]. All of the above indicate that patients with CAI alter their gait patterns. The findings of the current study support this notion and show another aspect of gait assessment. Patients with CAI have altered spatiotemporal gait patterns compared to a healthy control group including a lower walking velocity (~16%), lower cadence (~9%) and lower step length (~7%). Based on previous works and the results of the current work it may be concluded that patients with CAI alter their gait patterns and choose protective gait mechanisms strategies as part of their adaptations to ankle instability sensation during locomotion.

There is some evidence of sensorimotor deficits and balance impairments in patients with functional ankle instability [4,31-33], which may also be expressed in dynamic gait metrics. In a systematic review including a meta-analysis [34], Hiller et al. concluded that static measures of postural stability were significantly different between patients with CAI and healthy controls. Patients also described deficits in the performance of more strenuous stability challenges, such as jump tests. The rationale for the current work was that by assuming that patients with CAI have sensorimotor and postural control deficits as well as balance impairment in both static and dynamic conditions, these will also be manifested in spatiotemporal gait metrics. Indeed, the results of the current work show significant deviations in spatiotemporal gait patterns of patients with CAI compared to healthy controls. An interesting finding was the differences in base of support. Patients with CAI walked with a significantly wider base of support compared to healthy participants. It may be postulated that a wider base of support is a walking strategy adopted by patients with CAI to increase stability while walking. However this should be further investigated and validated. The base of support was ~43% wider, and the single limb support, which is another parameter that may reflect stability capabilities, was ~3.5% shorter than those parameters in the control group. Although significant differences were found between groups in both the base of support and single limb support, the clinical relevance of these results cannot be determined. More specifically, single limb support values were within the lower threshold of normal values (38.5%) [35]. We believe, however that this is the first report of these findings during functional tests in patients with CAI, and it may be assumed that they are related to impairment in dynamic balance during gait. It is reasonable to assume that if patients have an ankle instability sensation during walking they will choose a compensatory strategy that will reduce the single limb support, which is a phase where the entire body weight is on one limb, and will increase the double limb support phase. This strategy reduces the sole demand from the unstable ankle to maintain balance while walking. Furthermore, increasing the base of support may also be a compensatory strategy of patients with CAI. Increasing the walking base of support will allow better stability while walking and is assumed to reduces the demand to maintain stability from the unstable ankle. On the basis of the results, it may be assumed that patients with CAI have a distinct deficit in their balance that can be quantified. A test of base of support during gait may serve as a valid tool to assess such imbalance and may support the findings of the clinical evaluation of the patient.

Velocity, step length, stride length, single limb support and double limb support scores were all found in correlation with subjective pain and function scores (i.e., the SF-36 MCS and the SF-36 PCS). This suggests that these gait metrics could be utilized for quantitative evaluation and support of the self-reported pain and function of patients with CAI. Adding an objective assessment of the patient’s functional condition, alongside the patient’s subjective report may draw a better clinical picture that will help determine the optimal treatment. Furthermore, using both objective and subjective evaluation methods may help in the assessment of treatment outcomes. Recently single limb support was reported to be a valuable and sensitive parameter that is directly associated to the severity of knee osteoarthritis [14,36]. Future studies should further examine the correlations between the base of support and single limb support to the symptomatic severity of CAI in order to determine if they are sensitive in detecting different severity levels of CAI.

There are several limitations to the current study. Firstly, only spatiotemporal gait data were gathered. Three-dimensional gait analyses would provide more information on the kinematics and kinetics of the lower limb in patients with CAI as well as greater accuracy in determining dynamic instability during gait [37]. Those examinations, however, are relatively complex and costly. We sought to define objective gait metrics that can be easily obtained with relatively low cost, thus making them ideal in clinical practice. Another limitation of this study is that the data were collected retrospectively from the database of one therapy center. Since all patients in this database were referred for treatment, there might have been a selection bias.

## Conclusions

In conclusion, there were significant differences in the spatiotemporal gait profiles of patients with CAI and healthy controls. Specifically, all the tested parameters related to stability were worse in patients with CAI, which may reflect a gait strategy adopted by the patients to cope with their instability sensation.

Furthermore, worse subjective disease severity (as determined by pain and function self-evaluation questionnaires) were associated with more severe objectively identified gait abnormalities. This may suggest that using spatiotemporal gait assessment may add additional important information, alongside the clinical assessment of the patient, and may help in objectively quantify the functional condition of the patient. This assumption should be further examined and validated in future research.

### Consent

This was a retrospective analysis of an existing database, therefore patient's consent was not obtained. The Institutional Review Board approved the use of data without having patients signing consent as long as privacy and anonymity is fully kept.

## Abbreviations

CAI: Chronic ankle instability
MI: Mechanical instability
FI: Functional instability
BMI: Body mass index
GC: Gait cycle
QoL: Quality of life
PCS: Physical component summary
MCS: Mental component summary
